# Is Unidentified Cytopenia Truly Unidentified? Or Are We Overlooking Clonality?

**DOI:** 10.3390/medicina62050868

**Published:** 2026-05-01

**Authors:** Elcin Erdogan Yucel, Hale Bulbul Dilmen, Ozge Ozer Kaya, Zehra Narli Ozdemir, Merve Kakci, Bahriye Celik, Mustafa Kemal Yeniay, Fatma Keklik Karadag, Aybuke Olgun, Taha Resid Ozdemir, Cengiz Ceylan, Oktay Bilgir, İnci Alacacıoğlu

**Affiliations:** 1 Department of Hematology, Izmir City Hospital, University of Health Sciences, Izmir 35535, Turkey; halebulbul95@yahoo.com (H.B.D.); zehra.narliozdemir@sbu.edu.tr (Z.N.O.); b.celik_2008@hotmail.com (B.C.); mustafakemalyeniay@gmail.com (M.K.Y.); aybukeh.olgun@gmail.com (A.O.); cengiz.ceylan@sbu.edu.tr (C.C.); obilgir@hotmail.com (O.B.); 2 Genetic Diagnosis Center, University of Health Sciences, Izmir City Hospital, Izmir 35540, Turkey; drozgeozer@gmail.com (O.O.K.); dr.tahaoz@gmail.com (T.R.O.); 3 Department of Hematology, Dokuz Eylul University, Izmir 35210, Turkey; merve.kakci@deu.edu.tr (M.K.); inci.alacacioglu@deu.edu.tr (İ.A.); 4 Department of Hematology, Ege University, Izmir 35040, Turkey; fatma_keklik86@hotmail.com

**Keywords:** cytopenia, clonal hematopoiesis, sequence analysis, myelodysplastic syndromes

## Abstract

*Background and Objectives*: Peripheral cytopenia occurs in approximately 2% of the population; however, in up to 0.9%, no cause is identified by conventional tests. Next-Generation Sequencing (NGS) detects somatic variants consistent with clonal hematopoiesis (CH). We aimed to determine the prevalence of CH and pre-myelodysplastic syndrome (pre-MDS) using a 51-gene panel with histopathological assessment. *Materials and Methods*: Bone marrow samples from 96 consecutive patients evaluated for cytopenia were retrospectively analyzed for genetic alterations. *Results*: In the overall cohort (*n* = 96), the median follow-up was 8.1 months (range, 1–20). A total of 37 (39%) out of 96 patients were diagnosed with idiopathic cytopenia of undetermined significance (ICUS), 9 (9%) with clonal cytopenia of undetermined significance (CCUS), 9 (9%) with clonal cytopenia and monocytosis of undetermined significance (CCMUS), 34 (36%) with myelodysplastic syndrome (MDS), and 7 (7%) with myelodysplastic/myeloproliferative neoplasm (MDS/MPN). Among 41 patients in whom no cytogenetic abnormalities were detected by fluorescence in situ hybridization (FISH), somatic variants were identified by NGS. In CCUS, 88% of patients had a single variant, most commonly *ASXL1* (44%), followed by *TET2* (22%). In CCMUS, *ASXL1* and *DNMT3A* (each 25%) were the most frequent variants. The mean variant allele frequency (VAF) was higher in MDS (33.4%) than in CCUS/CCMUS (13.6%). In MDS patients aged 60 years and older, a higher number of variants were found compared to patients aged less than 60 years (*p* = 0.028). *RUNX1* variants (n = 8) were associated with leukopenia (*p* = 0.012). Patients with *SRSF2* variants (n = 4) had significantly poorer progression-free survival (*p* = 0.001). *EZH2* and *SETBP1* variants were associated with inferior overall survival (*p* = 0.04 and *p* = 0.019, respectively). In MDS patients (n = 34), thrombocytopenia (plt < 100.000) was associated with shorter PFS (*p* = 0.005). *Conclusions*: Given that pre-MDS conditions are considered predictors of hematologic malignancies, conventional diagnostic workup may be insufficient to accurately identify these entities, whereas NGS provides significant additional diagnostic value.

## 1. Introduction

Peripheral cytopenia occurs in 2% of the general population, with prevalence increasing with age [1]. The most common causes include nutritional deficiencies, drug/toxin exposure, infection, inflammation, immunological disorders, and malignancies. However, the cause remains unexplained despite routine blood and/or bone marrow histopathological examinations in up to 0.9% of patients [1]. If the peripheral cytopenia cannot be explained by conventional tests and by concomitant comorbid diseases, this condition is named as ‘unexplained cytopenia (UC)’ [2]. Next-Generation Sequencing (NGS) has been utilized to investigate UC in combination with conventional cytogenetics, fluorescence in situ hybridization (FISH), and polymerase chain reaction (PCR). While variants strongly associated with myelodysplastic syndrome (MDS), acute myeloid leukemia (AML), and myeloproliferative neoplasms (MPN) have been identified, NGS can reveal various variants in up to 30% of patients with cytopenia [3]. The World Health Organization (WHO) Classification of Haematolymphoid Tumors, 5th edition (WHO-HAEM5) and the International Consensus Classification (ICC) of myeloid neoplasms and acute leukemias have developed the definitions of “clonal hematopoiesis (CH)”, “myeloid precursor lesions”, and “premalignant clonal cytopenia” for those with somatic variants who do not meet the diagnostic criteria for specific hematological disorders [4,5].

Although the definitions of “CH” and “potential prephases of MDS” (pre-MDS) have been introduced in the literature, the prognostic significance of these variants, their association with cytopenia and dysplasia, and diagnostic value remain unclear. Moreover, daily practice can present challenges to these definitions.

In this study, we aimed to determine the prevalence of CH detected by NGS, particularly among patients presenting with UC. We further classified patients with “idiopathic cytopenia of unknown significance” (ICUS), “clonal cytopenia of undetermined significance” (CCUS), and “clonal cytopenia and monocytosis of undetermined significance” (CCMUS), to define the impact of the mutational variations on the prognosis, clinical, and morphological properties, thereby contributing to the existing literature.

## 2. Materials and Methods

### 2.1. Patients and Samples

This retrospective cohort study included 96 consecutive patients referred to the hematology outpatient clinic between September 2023 and December 2024 due to peripheral cytopenia. All patients provided informed consent. The study was conducted in accordance with the ethical principles of the Declaration of Helsinki and approved by the local Institutional Ethics Committee (date: 8 October 2024; number: 2024/160).

Patients who were 18 years or older, had a cytopenia in at least one cell type for four months or longer, and underwent routine diagnostic evaluation including bone marrow aspiration/biopsy, cytogenetic and FISH analysis, and whose NGS panels were examined for myeloid malignancies, were included in the study. Those who had an active myeloid/lymphoid neoplasia, solid malignancy, chronic liver disease, chronic renal failure, connective tissue disorder/metabolic disorder that is symptomatic or under treatment, vitamin deficiency, or lacked sufficient data were excluded.

Cytopenia refers to the presence of anemia, neutropenia, and/or thrombocytopenia. Anemia is defined as a hemoglobin (Hb) level of less than 13 g/dL in males and less than 12 g/dL in females; neutropenia as an absolute neutrophil count (ANC) of less than 1.8 × 10^9^/L; and thrombocytopenia as a platelet count (PLTs) of less than 150 × 10^9^/L [1,2].

We classified pre-MDS entities based on WHO-HAEM5 and ICC 2022 criteria as clonal hematopoiesis (CH) of indeterminate potential (CHIP) and clonal monocytosis of undetermined significance (CMUS), clonal cytopenia of undetermined significance (CCUS), clonal cytopenia and monocytosis of undetermined significance (CCMUS), idiopathic cytopenia of unknown significance (ICUS), and myelodysplastic syndrome with myeloproliferative neoplasm (MDS/MPN). ICUS, CCUS, and CCMUS were defined as pre-MDS conditions in the study.

Dysplasia and morphological assessments were independently evaluated by three experienced hematology professors in accordance with WHO-HAEM5 and ICC criteria. In cases with borderline findings, a consensus diagnosis was established.

The graphical abstract was generated using ChatGPT (OpenAI, San Francisco, CA, USA; accessed in [March 2026]), a web-based generative AI tool without a fixed version number. The GenAI tool was not used for data analysis, data interpretation, generation of scientific results, or formulation of scientific conclusions.

### 2.2. Next-Generation Sequencing Analysis

#### 2.2.1. DNA Isolation

Genomic DNA isolation from bone marrow aspiration samples was performed using the Magna Pure 96 automated isolation system (Roche Diagnostics, Mannheim, Germany), following the manufacturer’s protocol. The concentration of the isolated DNA was measured with a Qubit device using the iQuant dsDNA HS Assay Kit (INVITROGEN-QUBİT 4.0, Thermo Fisher Scientific, Waltham, MA, USA).

#### 2.2.2. Library Preparation and Sequencing

For the preparation of the NGS library, we utilized the KAPA HyperPETE Hematology Hotspot Panel (Roche Sequencing Solutions, Pleasanton, CA, USA), the 1000018646-MGIEasy Duplex UMI Adapters Kit (MGI Tech Co., Ltd., Shenzhen, China), and 9211624001-KAPA HyperPETE Reagent Kits (Roche Sequencing Solutions, Pleasanton, CA, USA). In the final stage, we employed the 1000020570-MGIEasy Dual Barcode Circularization Module Kit (MGI Tech Co., Ltd., Shenzhen, China) to ensure compatibility with the MGI device (MGI-G400, MGI Tech Co., Ltd., Shenzhen, China). The device was then loaded with a cartridge suitable for operation on the MGI G400 device alongside the required number of samples. NGS was performed using a myeloid panel targeting genes commonly mutated in myeloid malignancies from bone marrow samples.

Encoding regions for *ABL1*, *ASXL1*, *BCOR*, *BCORL1*, *BRAF*, *CALR*, *CBL*, *CEBPA*, *CSF3R*, *CUX1*, *DDX41*, *DNMT3A*, *ETNK1*, *ETV6*, *EZH2*, *FBXW7*, *FLT3*, *GATA1*, *GATA2*, *GNAS*, *IDH1*, *IDH2*, *IKZF1*, *JAK1*, *JAK2*, *JAK3*, *KDM6A*, *KIT*, *KMT2A*, *KRAS*, *MAP2K1*, *MPL*, *NF1*, *NOTCH1*, *NPM1*, *NRAS*, *PHF6*, *PTPN11*, *RAD21*, *RUNX1*, *SETBP1*, *SF3B1*, *SRSF2*, *STAG2*, *STAT3*, *TET2*, *TP53*, *U2AF1*, *U2AF2*, *WT1*, and *ZRSR2* genes were targeted.

The prepared libraries were sequenced on the MGI-G400 platform using a 2 × 150 bp paired-end read strategy. Our target was an average coverage depth of over 1000×. The analysis was conducted following the bioinformatics workflow recommended by Roche Sequencing Solutions, Inc. This workflow includes data quality control, alignment, deduplication, variant calling, and evaluation of results. The variants identified were interpreted using the NAVIFY Mutation Profiler (https://eu.mutationprofiler.navify.com; accessed on 5 November 2025) platform. We set the minimum variant allele frequency (VAF) threshold for the analysis at 2%.

#### 2.2.3. Variant Interpretation

Variants were analyzed using the NAVIFY Mutation Profiler platform. A minimum variant allele frequency (VAF) threshold of 2% was applied. Given the high sequencing depth (>1000×), this threshold was selected to balance sensitivity for detecting low-frequency variants and specificity by minimizing potential sequencing artifacts, consistent with previous studies reporting thresholds between 1% and 5% in similar settings [2,3].

Common variants (minor allele frequency >1% in population databases such as gnomAD) were excluded, along with variants not expected to have functional impact, such as synonymous changes, unless predicted to affect splicing.

Variants were classified according to the guidelines of the American College of Medical Genetics and Genomics (ACMG) for germline variants and the joint recommendations of the Association for Molecular Pathology (AMP), American Society of Clinical Oncology (ASCO), and College of American Pathologists (CAP) for somatic variants. Reporting primarily focused on pathogenic and likely pathogenic variants; for somatic alterations, Tier I and Tier II variants were included.

In addition, variants previously reported in the literature to be associated with disease were also taken into consideration during interpretation and, when relevant, discussed in the clinical context, even if they did not fully meet pathogenicity or tier classification criteria.

All variants were evaluated based on overall sequencing quality metrics, and orthogonal validation was not routinely performed.

### 2.3. Statistical Analyses

Categorical variables were represented as numbers (n) and percentages (%), whereas continuous variables were presented as mean ± SD if parametric test assumptions were satisfied, and as median (minimum–maximum) if they were not. The Kolmogorov–Smirnov Test was employed to assess the normality of variable distributions. The association between two category variables was examined using the chi-square test or Fisher’s exact test. Student’s *t*-test was employed when the two independent means had a normal distribution, and the Mann–Whitney U Test when they did not. The Kruskal–Wallis test was employed to compare more than two independent groups that exhibited non-normal distribution.

Endpoints were overall survival (OS), progression-free survival (PFS), and leukemia-free survival (LFS). PFS was defined as the time from diagnosis to disease progression, death, or the most recent follow-up visit. OS was assessed from the date of diagnosis to the date of death or the most recent follow-up visit. LFS is defined as the time from diagnosis of pre-MDS clinical conditions (ICUS, CCUS, CCMUS) until progression to MDS, chronic myelomonocytic leukemia (CMML), or acute leukemia.

Survival analyses were performed using the Kaplan–Meier method and compared with the log-rank test. Multivariate analyses were conducted using Cox proportional hazards regression models to control for potential confounding factors. Age, sex, and variant status were included as predefined confounders in all models, irrespective of their statistical significance. Hazard ratios (HRs) with 95% confidence intervals (CIs) were reported. Survival analyses could not be performed in pre-MDS cases, as no deaths or progression occurred except in one patient.

The data analysis was performed using the Statistical Package for the Social Science (SPSS Inc., Chicago, IL, USA) version 24.0, and a *p* < 0.05 was considered significant.

## 3. Results

### 3.1. Baseline Characteristics and the Findings of the Diagnostic Workup

Data from 96 patients who met the predefined inclusion and exclusion criteria were retrospectively analyzed. The median age was 66 (range, 27–93), and 44 (46%) of the patients were female (Table 1). Baseline comorbidities were also evaluated. Among the 96 patients, 11 had diabetes mellitus, 14 had controlled hypertension, and 8 had coronary artery disease, while 29 were active smokers. These variables did not differ significantly among the pre-MDS, MDS, and MDS/MPN groups.

Following conventional diagnostic methods, NGS, bone marrow aspiration, and biopsy, 37 of 96 patients (39%) were diagnosed with ICUS, 9 (9%) with CCUS, 9 (9%) with CCMUS, 34 (36%) with MDS, and 7 (7%) with MDS/MPN.

Hemoglobin levels were higher in ICUS cases compared to MDS cases (median 10.9 g/dL vs. 9.1 g/dL; *p* < 0.005) (Table 1). Additionally, lymphocyte counts were found to be higher in ICUS patients compared to CCUS patients (median 1.74 × 10^9^/L vs. 1.15 × 10^9^/L; *p* < 0.005) (Table 1).

The WBC count in patients with MDS/MPN was significantly higher than in the ICUS group (median: 17.8 vs. 5.7 × 10^9^/L; *p* = 0.005) (Table 1). In contrast, monocyte and platelet counts were significantly lower in patients with MDS/MPN (n = 7) compared with the ICUS group (n = 37) (*p* = 0.005 and *p* < 0.001, respectively).

In patients with ICUS, single-lineage cytopenia (70%) was more common than bicytopenia or pancytopenia (*p* = 0.0009). In patients with MDS, pancytopenia was significantly more frequent compared with pre-MDS patients (*p* < 0.003). When cases were evaluated for the presence of dysplasia, megakaryocytic dysplasia was more frequent in patients with ICUS and CCUS compared with erythroid or myeloid dysplasia (*p* = 0.006 and *p* = 0.018, respectively). In contrast, the frequency of dysplasia across all three lineages was similar among patients with CCMUS, MDS, and MDS/MPN (Table 1).

### 3.2. Frequency and Spectrum of Variants

In the 37 patients with cytopenia who did not meet the morphological criteria for MDS, no diagnosis-related genetic variants were detected by NGS and FISH/cytogenetic analysis, and these patients were classified as ICUS based on diagnostic evaluations.

Cytogenetic abnormalities were detected by FISH in 9 patients, while 87 patients had negative FISH results. Variants were identified by NGS in 45 patients. Among 41 patients in whom no cytogenetic abnormalities were detected by FISH, variants were identified by NGS. Of these patients, 8 were diagnosed with CCUS, 8 with CCMUS, 7 with MDS/MPN, and 18 with MDS.

Among patients with variants detected by NGS (n = 45), the median variant allele frequency (VAF) was 25.7% (range, 1–87%). In patients with MDS, the median VAF was 33.4%. In contrast, the median VAF in patients with CCUS and CCMUS (n = 18) was lower than that observed in MDS patients. The distribution of gene variants and corresponding VAF values are presented in Table 2.

**Figure 1:**
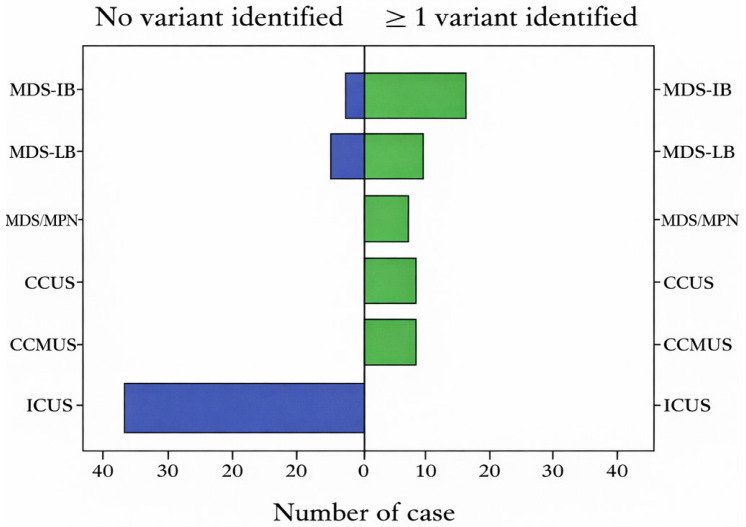
Bar graph representing the number of patients (n = 96) with pre-MDS, MDS, and MDS/MPN in which at least one variant was detected. ICUS, idiopathic cytopenia of unknown significance; CCUS, clonal cytopenia of undetermined significance; CCMUS, clonal cytopenia and monocytosis of undetermined significance; MDS-LB, myelodysplastic syndrome with low blast count; MDS-IB, myelodysplastic syndrome with increased blast count; MDS/MPN, myelodysplastic syndrome with myeloproliferative neoplasm.

**Figure 2:**
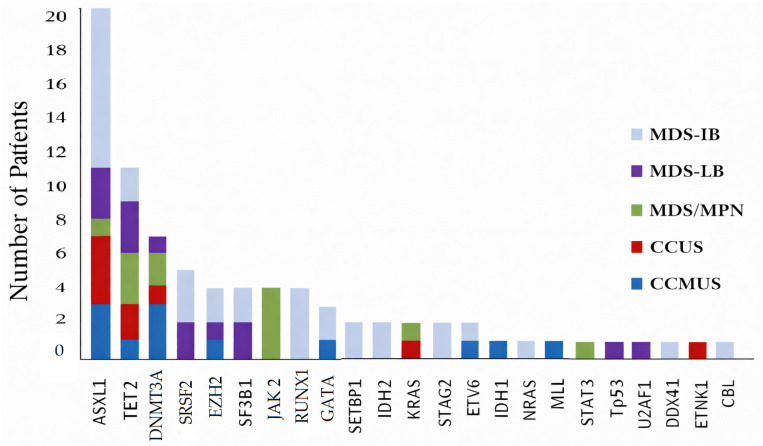
Number of patients carrying variants detected by NGS (n = 45). CCUS, clonal cytopenia of undetermined significance; CCMUS, clonal cytopenia and monocytosis of undetermined significance; MDS-LB, myelodysplastic syndrome with low blast count; MDS-IB, myelodysplastic syndrome with increased blast count; MDS/MPN, myelodysplastic syndrome with myeloproliferative neoplasm.

The variant frequency in pre-MDS, MDS, and MDS/MPN patients is shown in Figure 1. *ASXL1* (22.7%), *TET2* (12.5%), and *DNMT3A* (7.9%) were the most common variants in this patient group (Figure 2).

In pre-MDS, MDS, and MDS/MPN patients (n = 96), *ASXL1* (33%) and *DNMT3A* (18.5%) were the most frequently detected variants in patients with anemia (n = 54); *ASXL1* (42%) was the most frequently detected variant in patients with leukopenia (n = 24); and *ASXL1* (33%) and *TET2* (27%) were the most frequently detected variants in the patients with thrombocytopenia (n = 30).

### 3.3. Gene Variants Landscape in the CCUS Group

**Figure 3:**
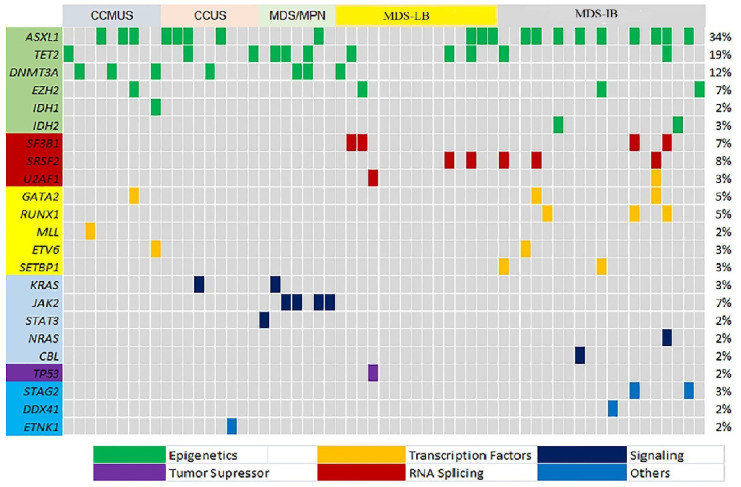
Variant landscape of bone marrow samples of 59 patients and subgroups of CCUS, CCMUS, MDS-LB, MDS-IB, and MDS/MPN. Each column represents a patient. Colors represent the type of variant. The percentage of patients with variants in each gene is presented on the right line. CCUS, clonal cytopenia of undetermined significance; CCMUS, clonal cytopenia and monocytosis of undetermined significance; MDS-LB, myelodysplastic syndrome with low blast count; MDS-IB, myelodysplastic syndrome with increased blast count; MDS/MPN, myelodysplastic syndrome with myeloproliferative neoplasm.

Among patients with CCUS, 88% harbored a single variant, most frequently in *ASXL1* (44%), followed by *TET2* (22%), *DNMT3A* (11%), *KRAS* (11%), and *ETNK1* (11%) (Figure 3). One patient (patient no. 17) was diagnosed with CCUS based on the presence of del (4q) and <10% erythroid dysplasia. Co-variants in *ASXL1* and *TET2* were detected in one patient (patient no. 12) (Figure 3). In eight patients with negative FISH results, NGS identified pathogenic variants, leading to a diagnosis of CCUS.

### 3.4. Gene Variant Landscape in the CCMUS Group

The most frequently detected variants in CCMUS patients were *ASXL1* (25%), *DNMT3A* (25%), and *TET2* (8.3%). In CCMUS, multiple variants in the same patients were common. One patient had *ASXL1*, *EZH2,* and *GATA* variants (patient no:7), while another had *DNMT3A*, *IDH1,* and *ETV6* (patient no 9) (Figure 3). In one patient, monocytosis was accompanied by *del11q*, but there was no significant dysplasia (patient no:8). Variants were detected by NGS in eight patients who had no abnormalities identified by FISH analysis, and these patients were subsequently diagnosed with CCMUS.

### 3.5. Gene Variant Landscape in the MDS Group

Thirty-four patients were diagnosed with MDS. In nine patients, no variants were detected by either NGS or FISH; however, the diagnosis was established based on bone marrow aspiration, biopsy findings, and clinical evaluation. Variants were identified exclusively by NGS in 18 patients and exclusively by FISH in 3 patients. In four patients, both somatic variants by NGS and cytogenetic abnormalities by FISH were detected.

In MDS patients aged 60 years and older, a higher number of variants was found compared to patients aged less than 60 years (OR: 0.91; 95% CI: 0.10–1.72; *p* = 0.028).

Nine patients (26%) had no variant, 24% had one variant, 26% had two variants, 24% had three variants in MDS (n = 34) patients (Table 1).

In myelodysplastic syndrome with increased blasts (MDS-IB) (n = 19), variant rate was 84%, while it was %67 in myelodysplastic syndrome with low blasts (MDS-LB) (n = 15) group.

The most common variants in MDS-LB patients were *ASXL1* (27.2%), *TET2* (27.2%), and *SRSF2* (18.1%) (Figure 2).

The most common variants in MDS-IB patients were *ASXL1* (23.6%), *RUNX1* (10.5%), and *SRSF2* (7.8%) (Figure 2). *RUNX1* variants (n = 8) were associated with leukopenia (*p* = 0.012).

Loss of 5q was detected in two patients with MDS-LB and one patient with MDS-IB. Loss of 7q was detected in two patients with MDS-LB (n = 1) and MDS-IB (n = 1). Loss of 20q was detected in a case with MDS-IB.

### 3.6. Gene Variant Landscape in the MDS/MPN Group

All MDS/MPN patients (n = 7) were aged 60 years or older. The most frequently detected somatic variant was *JAK2* (57%), identified in four patients. The *TET2* variant (42%) was detected in three patients, and *DNMT3A* (28%) in two patients (Figure 2). A combination of two variants was detected in five (71%) of the MDS/MPN patients (n = 7) (Table 1). In the MDS/MPN cohort, FISH analysis failed to demonstrate any detectable cytogenetic abnormalities.

### 3.7. Survival Analysis of the Entire Cohort

**Figure 4:**
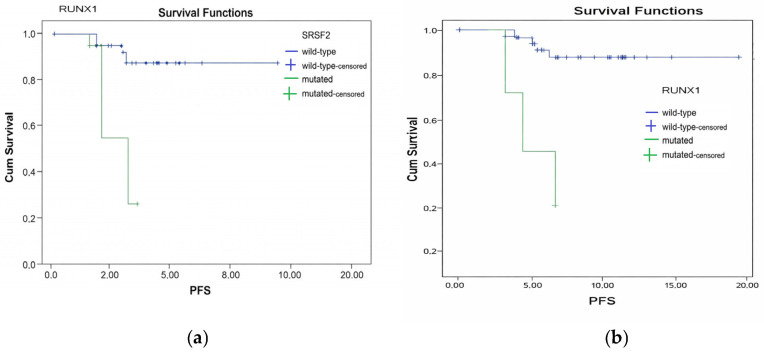
Progression-free survival (PFS) analysis of pre-myelodysplastic syndroma, myelodisplastic syndrome, and myelodysplastic syndrome with myeloproliferative neoplasm cohort (n = 96), according to presence of *SRSF2* (**a**) and *RUNX1* (**b**) variants. Univariate analysis showed patients with *SRSF2* and *RUNX1* variants had significantly inferior PFS (*p* = 0.001; *p* = 0.01, respectively).

For the entire cohort (n = 96), the median follow-up was 8.1 months (range, 1–20), the median PFS and OS were 7.14 months (range, 0–20) and 8.6 months (range, 1–20), respectively. In this patient group, patients with the *SRSF2* variant (n = 4) had significantly shorter PFS (HR: 18.2; 95% CI: 1.7–199; *p* = 0.001), while no significant difference was detected in OS (see Figure 4a and Table 3 which demonstrate PFS according to presence of SRSF2).

Patients with *RUNX1* variants (n = 4) had poorer PFS (HR: 4.75; 95% CI: 1.5–13.19; *p* = 0.01), but no significant difference was observed in OS (see Figure 4b and Table 3 which demonstrate PFS according to presence of RUNX1).

The mean LFS was 11.1 ± 3.8 months in patients with CCMUS (n = 9) and 8.5 ± 3.3 months in patients with CCUS (n = 9), with no statistically significant difference between the groups (*p* = 0.692).

An 86-year-old CCUS patient with a *DNMT3A* variant and 11% VAF transformed into MDS after 7.6 months. Death occurred due to infection during follow-up. Other CCUS and CCMUS patients developed no progression or death during their follow-up.

The mean PFS of MDS/MPN (n = 7) patients was 8.6 ± 3.04 months, and OS was 9.2 ± 2.5 months. In MDS-LB patients (n = 15), the mean PFS was 6.2 ± 3.08 months, and the mean OS was 8.1 ± 3.8 months. In MDS-IB patients (n = 19), the mean PFS was 4.7 ± 2.7 months, and the mean OS was 7.8 ± 3.2 months. No statistically significant difference was found between the groups (*p* > 0.05).

**Figure 5:**
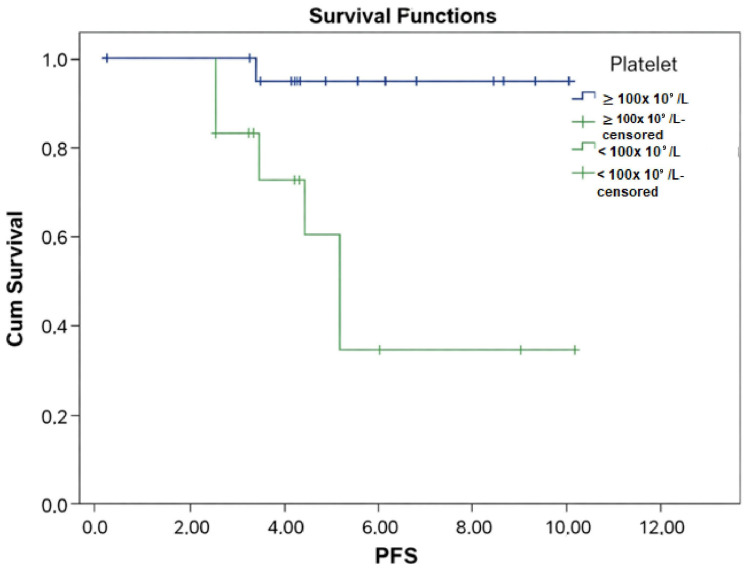
Progression-free survival (PFS) analysis of myelodysplastic syndrome cohort (n = 34) according to presence of trombocytopenia. Univariate analysis showed patients with thrombocytopenia had significantly inferior PFS (*p* = 0.005).

**Figure 6:**
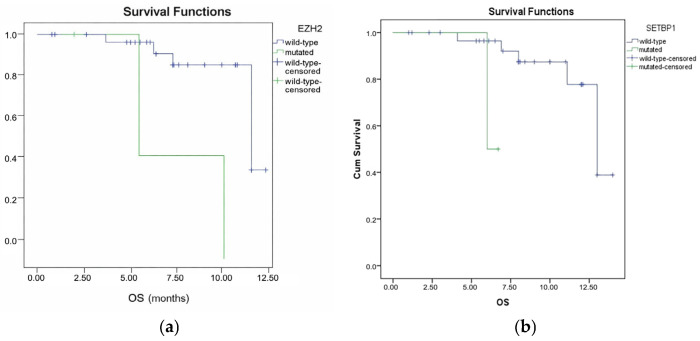
Overall survival (OS) analysis of myelodysplastic syndrome cohort (n = 34) according to presence of *EZH2* (**a**) and *SETBP1* (**b**) variants. Univariate analysis showed patients with *EZH2* and *SETBP1* variants had significantly inferior OS (*p* = 0.04; *p* = 0.019, respectively).

In MDS patients (n = 34), thrombocytopenia (platelet count < 100 × 10^9^/L) was associated with shorter PFS (HR: 2.85; 95% CI: 1.35–6.01; *p* = 0.005) (see Figure 5 and Table 3 which demonstrate PFS according to presence thrombocytopenia). *EZH2* and *SETBP1* variants correlated with inferior OS (*p* = 0.04, *p* = 0.019) (see Figure 6a,b, and Table 3 which demonstrate OS according to presence of *EZH2* and *SETBP1* retrospectively).

In summary, analysis of variant frequency and spectrum demonstrated that clonal states (CCUS/CCMUS) were identified in a substantial proportion of patients with UC. The most frequently detected variants in the pre-MDS group included *ASXL1*, *TET2*, and *DNMT3A*. Clinico-genetic correlations were observed, particularly for the association of *ASXL1* variants with multilineage cytopenia and dysplasia, suggesting a potentially more aggressive phenotype even at early disease stages. From a prognostic perspective, *SRSF2* and *RUNX1* variants were associated with inferior progression-free survival, while thrombocytopenia (<100 × 10^9^/L) also emerged as a significant adverse prognostic factor in MDS patients. Furthermore, a significant difference in median variant allele frequency (VAF) between CCUS/CCMUS and MDS patients supports the use of VAF as an additional diagnostic biomarker.

## 4. Discussion

To diagnose MDS, it is essential to identify at least one persistent cytopenia along with the presence of at least 10% morphological dysplasia in one cell series, with only rare exception harboring MDS-defining certain genetic abnormalities [5]. However, some patients investigated for UC do not meet these criteria, leading to their classification as potential prephases of MDS (pre-MDS). Research has shown that pre-MDS conditions can evolve into other hematological neoplasms or be associated with non-hematological diseases [6]. Therefore, it is crucial to understand pre-MDS and CH using advanced genetic techniques, such as NGS for effective patient monitoring. In this study, the genetic variation profile of 96 patients investigated for cytopenia in the hematology clinic was examined using NGS. Of these patients, 18 (33%) were diagnosed with CCUS and CCMUS, and 37 (67%) with ICUS. Cargo et al. analyzed 1485 patients with non-diagnostic bone marrow evaluations. Without distinguishing between CCUS and CCMUS, they detected CCUS in 26.9% of cases [7]. Although our patient group was relatively small, we detected CCUS/CCMUS at a similar frequency.

Patients in the ICUS group had higher levels of hemoglobin and lymphocyte, with cytopenia primarily observed in a single series. This finding aligns with the HUMARA study, which reported single series cytopenia in 66% of ICUS patients [8]. In MDS patients, pancytopenia and dysplasia in multiple series were more common, consistent with the literature [9]. Nielsen et al. reported higher inflammatory cytokines in ICUS and CCUS patients compared to healthy individuals but did not find a significant difference in lymphocyte count between patient groups [10]. To gain further insights, additional studies with larger patient cohorts are needed, utilizing flow cytometry to analyze lymphocyte subtypes while simultaneously examining cytokine profiles.

The most frequently detected somatic variants in pre-MDS and MDS patients were in the *ASXL1* and *TET2* genes. In our study, we observed a marked increase in concurrent cytopenia in the erythroid, myeloid, and megakaryocyte series in the presence of the *ASXL1* gene variant. The *ASXL1* gene encodes a protein involved in the epigenetic regulation of gene expression that is mutated in 10–20% of MDS cases [11]. In the present study, the detection of *ASXL1* variants in 22.7% of the pre-MDS group supports the notion that this variant plays a significant role not only in diagnosed MDS patients but also in pre-MDS stages. Similarly, *TET2* variants are reported to occur in approximately 15% of myeloid malignancies and in up to 30% of MDS patients [12]. In our cohort, the frequency of *TET2* variants was found to be 12.5% in pre-MDS patients. Although this rate is lower than the upper limit in the literature, it suggests that *TET2* variants may be present at a significant rate in preclinical stages and may be associated with clinical outcomes.

*ASXL1* and *TET2* were the most frequently detected variants in CCUS patients. Xie and colleagues developed the ‘Clonal Cytopenia Risk Score (CCRS)’ model to predict prognosis in CCUS patients [13]. This model categorizes patients into three groups—low, intermediate, and high risk—based on risk factors such as the presence of two or more variants, a platelet count of less than 100 × 10^9^/L, and variants in splice genes (*SF3B1*, *SRSF2*, *U2AF1*, etc.). In our cohort, 55% of CCUS/CCMUS patients (n = 11) were in the low-risk group, 28% (n = 5) in the intermediate-risk group, and 11% (n = 2) in the high-risk group. Although a significant relationship with LFS could not be demonstrated due to the limited number of patients and short duration of follow-up, our study contributes to the literature as one of the few studies directly testing the practical feasibility of the CCRS model.

In the literature, it has been reported that the *DNMT3A* variant in CCUS patients is generally associated with a better prognosis; conversely, splicing genes or MN-like variants carry a higher risk of progression [14]. In our study, an 80-year-old CCUS patient with a *DNMT3A* variant progressed to MDS within approximately 7.6 months, followed by infection-related death. However, it should be considered that this outcome may be related to the patient’s advanced age and comorbid conditions. This finding suggests that *DNMT3A* variants may not always follow a benign clinical course and highlights the need to evaluate the clinical impact of variants in the context of patient-specific characteristics.

CCMUS, defined in the 2022 ICC, is defined as a precursor condition that is characterized by the coexistence of variants associated with clonal hematopoiesis and persistent monocytosis, but that does not meet the criteria for CMML [5]. A large-scale study based on UK Biobank (UKB) data showed that CCMUS cases occur at a rate of 0.4% in the general population and that the 10-year risk of myeloid neoplasm (MN) progression reaches up to 18.6% [15]. In this study, the most frequently observed driver variants in CCMUS patients were *TET2* (37.6%), *SRSF2* (16.3%), and *DNMT3A*, with *TET2* and *SRSF2* variants in particular being associated with an increased risk of progression. In our patient group, the most frequently detected variant in individuals diagnosed with CCMUS was *ASXL1* (25%), followed by *DNMT3A* (25%) and *TET2* (8.3%). In this respect, our study differs from UKB data in terms of genetic variant distribution. In particular, the more frequent observation of dysplasia and cytopenia findings in individuals with the *ASXL1* variant suggests a possible effect of this variant on the CCMUS phenotype. Dysplasia was detected in three series at a level of less than 10% in all our CCMUS cases, and dysplasia and cytopenia findings were more pronounced in cases carrying two variants. The absence of MN progression in any patient during the follow-up period, although requiring careful interpretation due to the small sample size, highlights the importance of a detailed evaluation of the relationship between variant type and clinical phenotype.

In our study, the mean VAF value for somatic variants was estimated to be 33.4% in MDS patients, whereas this rate was 13.6% in CCUS and CCMUS patients. This significant difference suggests that the size of variant-carrying clones may be valuable in elucidating the etiology of cytopenia and in disease classification. In particular, the observation of low VAF rates in preclinical conditions, such as CCUS/CCMUS, and high VAF rates in more progressive neoplasms, such as MDS, indicates that VAF could be used as a prognostic marker.

Zheng et al. similarly reported higher VAF values in MDS patients compared to CCUS and they emphasized that this difference is significant in terms of diagnosis and prognosis [16]. On the other hand, in a large-scale study conducted by the Mayo Clinic, the mean VAF was found to be 40% in the MDS group and 32.5% in cytopenic cases that did not meet the morphological and genetic criteria for MDS, and this difference did not reach statistical significance (*p* = 0.07) [17]. This may have resulted from the heterogeneous patient group in the study, as well as from the diagnostic boundaries not being sufficiently clear clinically.

In contrast, the patient selection and molecular subgrouping in our study are more uniform; the difference between the low VAF values in the CCUS and CCMUS subgroups and the significant increase in the MDS group are both statistically significant and clinically interpretable. In this respect, our study demonstrates that VAF may be a biomarker with not only diagnostic but also prognostic discriminatory power, thereby contributing to the literature.

Beyond VAF-based comparisons, recent studies have also highlighted the broader clinical utility of Next-Generation Sequencing (NGS) in the evaluation of UC. In particular, targeted gene panel sequencing has been shown to improve the distinction between clonal and non-clonal cytopenias and to contribute to more accurate diagnostic classification [16]. Moreover, molecular profiling may provide additional prognostic information in patients with cytopenia and suspected MDS [17]. In this context, our findings further support the role of NGS in identifying clonal alterations in patients with UC, enabling a more refined classification of pre-MDS conditions and potentially contributing to improved risk stratification and follow-up strategies.

In our study, a significantly higher number of variants were detected in MDS patients aged 60 years and older compared to those aged less than 60 years. This finding is consistent with previous literature showing an increase in variant burden with age [18].

Hogg et al. reported that *TET2* (21%), *ASXL1* (18.2%), and *SF3B1* (12.6%) were the most common variants in MDS. [18] In our study, *ASX1*, *TET2*, and *SRSF2* were the most frequently detected. *RUNX1* variant was present in 10.5% of MDS-IB patients, and leukemia was also present in patients with *RUNX1* variant. The literature data report that *RUNX1* variants, seen in 10% of MDS patients, are associated with high risk and poor prognosis [19]. In our study, MDS patients with *RUNX1* variants exhibited a poorer prognosis. We also demonstrated that MDS-IB patients have more variants, consistent with the literature.

In this study, the *TET2* variant was detected in 42% of MDS/MPN patients. In another study, the frequency of mutant *TET2* was reported as 38.6% in 44 MDS/MPN-unclassifiable patients [20].

In our study, the presence of thrombocytopenia was found to be associated with poor PFS, consistent with the literature [21]. Furthermore, *EZH2* and *SETBP1* mutant MDS patients had lower OS compared to wild-type MDS patients. Hou et al. followed MDS patients with the *SETBP1* variant for 43.9 months and demonstrated that AML transformation was more frequent and associated with poorer OS. [22] Another study investigating 1774 patients reported that *EZH2* variant was observed in 4.7% of MDS patients and was associated with poor OS [23]. However, given the small number of patients with *SRSF2*, *EZH2*, and *SETBP1* variants in our cohort, these findings should be interpreted with caution and considered exploratory.

Based on our findings, a potential clinically relevant gene signature may be proposed to aid in differential diagnosis and risk stratification. Variants in epigenetic regulators such as *ASXL1*, *TET2*, and *DNMT3A* were predominantly observed in clonal cytopenias (CCUS/CCMUS), whereas variants in splicing factors (e.g., *SRSF2*) and transcriptional regulators (e.g., *RUNX1*) were associated with inferior PFS. In addition, higher VAF levels were more characteristic of MDS compared to pre-MDS conditions. Taken together, the integration of variant type, number of variants, and VAF may represent a practical framework for distinguishing pre-MDS conditions from overt myeloid neoplasms and for guiding clinical decision-making.

Our study has some limitations. We excluded patients with clonal hematopoiesis of indeterminate potential (CHIP) and clonal monocytosis of undetermined significance (CMUS) because the focus was on patients with cytopenia. One of the main limitations of our study is the relatively short median follow-up duration (8.1 months) and the limited number of events, both of which restrict the ability to draw definitive conclusions regarding long-term outcomes. Therefore, the survival analyses and associated prognostic findings should be interpreted with caution and considered as preliminary and hypothesis-generating rather than definitive. In addition, some clinical parameters such as performance status, treatment regimens, inflammatory markers, transfusion data, and detailed symptom profiles were not consistently available due to the retrospective nature of the study. However, our cohort represents a relatively homogeneous UC population evaluated using uniform diagnostic criteria and NGS-based molecular profiling, which strengthens the internal validity of our baseline findings. Longer follow-up will be valuable to further clarify the prognostic impact of detected variants and the risk of progression.

In addition, although co-variants were identified, a formal analysis of gene–gene interactions was not performed. This was mainly due to the relatively low frequency of specific variants, which limited the statistical power for such analyses. Future studies with larger cohorts are needed to better explore potential interactions between variants.

## 5. Conclusions

The increasing use of NGS has significantly changed the approach to cases of cytopenia of unknown cause. Given that pre-MDS conditions are thought to be predictive of hematological malignancies, it is clear that the detection of CH is important in the follow-up and treatment of patients. There is a need for multi-center studies investigating specific variants associated with pre-MDS and the effects of these variants on clinical course and prognosis.

## Figures and Tables

**Table 1:** Clinical characteristics of patients with pre-MDS, MDS, and MDS/MPN (n = 96).

Variable	Overall	ICUS	CCUS	CCMUS	MDS	MDS/MPN
n (%)	96	37 (39)	9 (9)	9 (9)	34 (36)	7 (7)
Sex (n/%)						
Male	52 (54)	16 (43)	6 (66)	5 (55)	22 (68)	3 (43)
Female	44 (46)	21 (57)	3 (34)	4 (45)	12 (32)	4 (57)
Age, years	66 (27–93)	64.2 (28–93)	63.3 (27–85)	72.8 (56–86)	67.8 (34–87)	66.5 (61–83)
WBC count, ×10^9^/L	6.6 (1.6–62.2)	5.7 (1.6–12.9)	4.7 (3.13–6.5)	6.54 (4.5–13.2)	7.3 (1.18–55.1)	17.8 (3.4–62.2)
ANC count, ×10^9^/L	3.9 (0.18–55.7)	3.19 (0.18–8.6)	3.04 (1.54–4.8)	3.57 (1.3–9.6)	4.52 (0.28–43.6)	13.2 (1.8–55.7)
Hemoglobin, g/dL	10.1 (5.8–15.5)	10.9 (6.3–14.5)	9.58 (7.1–12.9)	11 (8.1–13.6)	9.1 (5.8–13.10)	11 (8.6–15.50)
RBC count, ×10^12^/L	3.3 (2.6–5.8)	3.52 (2.7–5.5)	3.15 (2.9–4.8)	3.4 (2.6–5.2)	2.9 (2.6–5.1)	3.5 (3–5.8)
Platelet count, ×10^9^/L	195 (19–520)	239.5 (38–672)	220.3 (130–345)	305 (81–520)	154.7 (19–456)	107.1 (61–187)
Monocyte count, ×10^9^/L	0.58 (0–4.9)	0.55 (0.19–1.08)	0.37 (0.24–0.47)	0.7 (0.50–1.10)	0.63 (0–3.2)	0.44 (0.12–4.9)
Lymphocyte count, ×10^9^/L	1.77 (0.58–28)	1.74 (0.58–3.74)	1.15 (0.84–1.55)	3.58 (1–28)	1.83 (0.72–7.57)	2.22 (1.17–4.23)
Number of cytopenia (n/%)						
1	47 (49)	26 (70)	2 (22)	6 (67)	11 (32)	2 (29)
2	32 (33)	9 (24)	6 (67)	3 (33)	10 (30)	4 (58)
3	17 (18)	2 (6)	1 (11)	0	13 (38)	1 (13)
Number of variants (n/%)						
0	46 (48)	37	0	0	9 (26)	0
1	24 (25)	0	8 (88)	6 (67)	8 (24)	2 (29)
2	16 (17)	0	1 (12)	1 (11)	9 (26)	5 (71)
≥3	10 (10)	0	0	2 (22)	8 (24)	0
Dysplasia < %10 (Yes/No)						
Erythroid	33/63	15/22	1/8	4/5	10/24	3/4
Myeloid	25/71	13/24	2/6	2/7	6/28	2/5
Megakaryocytic	43/53	23/14	5/4	3/6	10/24	2/5
Overt dysplasia (Yes/No)						
Erythroid	8/88	-	-	-	7/27	1/6
Myeloid	15/66	-	-	-	13/21	2/5
Megakaryocytic	20/76	-	-	-	18/16	2/5

Data are presented as median (range) unless otherwise specified. ICUS, idiopathic cytopenia of unknown significance; CCUS, clonal cytopenia of undetermined significance; CCMUS, clonal cytopenia and monocytosis of undetermined significance; RBC, red blood cell; MDS, myelodysplastic syndrome; MDS/MPN, myelodysplastic syndrome with myeloproliferative neoplasm; WBC, white blood cells; ANC, absolute neutrophil count; overt dysplasia (≥10% dysplasia in the erythroid, myeloid or megakaryocytic lineage). No significant differences were found in age and gender among patients with ICUS, CCUS, and CCMUS, which were assessed as pre-MDS clinical conditions (*p* > 0.05).

**Table 2:** Distribution of gene variants and variant allele frequency (VAF) according to diagnosis (n = 45).

Gene	Number of Patients	VAF (%) Median (Range)	Diagnosis (Number of Patients)
*SF3B1*	4	41 (12–46)	MDS-LB (n = 2) MDS IB (n = 2)
*ASXL1*	20	24 (2.6–100)	CCMUS (n = 3); CCUS (n = 4); MDS/MPN (n = 1); MDS-LB (n = 3); MDS-IB (n = 9)
*EZH2*	4	18 (2.2–48)	CCMUS (n = 1); MDS-LB (n = 1); MDS-IB (n = 2)
*GATA1*	3	13 (10–22)	CCMUS (n = 1); MDS-IB (n = 2)
*SRSF2*	5	41 (33–46)	MDS-LB (n = 2); MDS-IB (n = 3)
*SETBP1*	2	18.5 (13–24)	MDS-IB (n = 2)
*TET2*	11	34 (3.6–76)	CCMUS (n = 1); CCUS (n = 2); MDS/MPN (n = 3), MDS-LB (n = 3); MDS-IB (n = 2)
*DNMT3A*	7	11 (1–60)	CCMUS (n = 3); CCUS (n = 1); MDS/MPN (n = 2); MDS-LB (n = 1)
*IDH1*	1	38	CCMUS (n = 1)
*IDH2*	2	33.5 (8.6–40)	MDS-IB (n = 2)
*NRAS*	1	23 (14–43)	MDS-IB (n = 1)
*KRAS*	2	36 (2.1–41)	CCUS (n = 1); MDS/MPN (n = 1)
*MLL*	1	21	CCMUS (n = 1)
*JAK2*	4	10 (2–87)	MDS/MPN (n = 4)
*CALR*	1	21 (8–34)	MDS/MPN (n = 1)
*STAT3*	1	13 (12–14)	MDS/MPN (n = 1)
*RUNX1*	4	38 (13–56)	MDS-IB (n = 4)
*TP53*	2	34 (47–72)	MDS-LB (n = 2)
*U2AF1*	1	70	MDS-LB (n = 1)
*ETV6*	2	5.7 (5.2–6.2)	CCMUS (n = 1); MDS-IB (n = 1)
*DDX41*	1	33	MDS-IB (n = 1)
*ETNK1*	1	42.5 (40–45)	CCUS (n = 1)
*CBL*	1	14	MDS-IB (n = 1)
*STAG2*	2	62 (37–87)	MDS-IB (n = 2)

Some patients harbored more than one variant. VAF, variant allele frequency; CCUS, clonal cytopenia of undetermined significance; CCMUS, clonal cytopenia and monocytosis of undetermined significance; MPN, myeloproliferative neoplasm. MDS-LB (myelodysplastic syndrome with low blasts): bone marrow blasts <5%. MDS-IB (myelodysplastic syndrome with increased blasts): bone marrow blasts ≥5%.

**Table 3:** Multivariate Cox proportional hazards regression analysis for progression-free survival (PFS) and overall survival (OS).

Variable	HR	95% CI	*p*-Value
PFS			
Age (years)	0.97	0.9–1.02	0.31
Sex (male vs. female)	1.1	0.21–6.5	0.84
Thrombocytopenia ^&^ (<100 × 10^9^/L)	2.85	1.35–6.01	0.005 *
*SRSF2* mutant vs. wild	18.2	1.7–199	0.001 *
*RUNX1* mutant vs. wild	4.75	1.5–13.9	0.01 *
OS			
Age (years)	1.0	0.95–1.12	0.42
Sex (male vs. female)	1.47	0.2–10.5	0.69
Thrombocytopenia (<100 × 10^9^/L)	1.1	0.14–7.20	0.98
*EZH2*^ &^ mutant vs. wild	9.2	1.5–56.6	0.04 *
*SETBP1*^ &^ mutant vs. wild	12.9	0.8–207.6	0.019 *

HR: hazard ratio; CI: confidence interval. Statistically significant *p*-values are indicated with an asterisk *. Variables marked with “&” indicate analyses restricted to patients with MDS.

## Data Availability

The data that support the findings of this study are available from the corresponding author upon reasonable request.

## Abbreviations

The following abbreviations are used in this manuscript:

WHO-HAEM5	The World Health Organization of Hematolymphoid Tumors Version 5
ICC	The International Consensus Classification
NGS	Next-Generation Sequencing
FISH	Fluorescence in situ hybridization
PCR	Polymerase Chain Reaction
AML	Acute Myeloid Leukemia.
VAF	Variant Allele Frequency
CCUS	Clonal Cytopenia of Undetermined Significance
CCMUS	Clonal Cytopenia and Monocytosis of Undetermined Significance
MPN	Myeloproliferative Neoplasm
MDS-LB	Myelodysplastic Syndrome with Low Blasts
MDS-IB	Myelodysplastic Syndrome With İncreased Blasts
PFS	Progression-Free Survival
OS	Overall Survival
Pre-MDS	Potential Prephases of MDS
